# Management of Accidental Laboratory Exposure to *Burkholderia pseudomallei* and *B. mallei*

**DOI:** 10.3201/eid1407.071501

**Published:** 2008-07

**Authors:** Sharon J. Peacock, Herbert P. Schweizer, David A.B. Dance, Theresa L. Smith, Jay E. Gee, Vanaporn Wuthiekanun, David DeShazer, Ivo Steinmetz, Patrick Tan, Bart J. Currie

**Affiliations:** * Mahidol University, Bangkok, Thailand; † Colorado State University, Fort Collins, Colorado, USA; ‡ Health Protection Agency (South West), Plymouth, UK; § Centers for Disease Control and Prevention, Atlanta, Georgia, USA; ¶ US Army Medical Research Institute of Infectious Diseases, Fort Detrick, Maryland, USA; # Universität Greifswald, Greifswald, Germany; **Genome Institute of Singapore, Singapore; †† Menzies School of Health Research and Royal Darwin Hospital, Darwin, Northern Territory, Australia

**Keywords:** *Burkholderia pseudomallei*, *B. mallei*, glanders, melioidosis, post-exposure prophylaxis, online report

The gram-negative bacillus *Burkholderia pseudomallei* is a saprophyte and the cause of melioidosis. Natural infection is most commonly reported in northeast Thailand and northern Australia (*1*,*2*) but also occurs in other parts of Asia, South America, and the Caribbean (*3*). Melioidosis develops after bacterial inoculation or inhalation, often in relation to occupational exposure in areas where the disease is endemic. Clinical infection has a peak incidence between the fourth and fifth decades (*1*,*2*); with diabetes mellitus, excess alcohol consumption, chronic renal failure, and chronic lung disease acting as independent risk factors (*4*). Most affected adults (≈80%) in northeast Thailand, northern Australia, and Malaysia have >1 underlying diseases (*2*,*5*,*6*). Symptoms of melioidosis may be exhibited many years after exposure, commonly in association with an alteration in immune status (*1*,*2*). Manifestations of disease are extremely broad ranging and form a spectrum from rapidly life-threatening sepsis to chronic low-grade infection. A common clinical picture is that of sepsis associated with bacterial dissemination to distant sites, frequently causing concomitant pneumonia and liver and splenic abscesses. Infection may also occur in bone, joints, skin, soft tissue, or the prostate (*1*,*2*,*5*). The clinical symptoms of melioidosis mimic those of many other diseases; thus, differentiating between melioidosis and other acute and chronic bacterial infections, including tuberculosis, is often impossible. Confirmation of the diagnosis relies on good practices for specimen collection, laboratory culture, and isolation of *B. pseudomallei*. The overall mortality rate of infected persons is 50% in northeast Thailand (35% in children) (*1*) and 19% in Australia (*2*).

*B. pseudomallei* has been designated a select agent by the US Centers for Disease Control and Prevention (CDC) (www.cdc.gov/od/sap). Interest in this organism has been fueled by the establishment of Regional Centers of Excellence across the United States funded by the National Institutes of Health (NIH)/National Institute of Allergy and Infectious Diseases for research into emerging infectious diseases and biothreat organisms. The number of investigators who are working on strains of *B. pseudomallei* is growing, and research laboratories require clearly defined readiness guidelines in the event that 1 or more persons require postexposure prophylaxis (PEP). Workers in clinical diagnostic laboratories may also be unwittingly exposed to *B. pseudomallei* before its identity is recognized, as exemplified by recent reports (*7*,*8*).

Two previously described cases of laboratory-acquired melioidosis illustrate the practices that led to exposure and the time lapse from exposure to onset of symptoms. The first case-patient was a 48-year-old laboratory worker who cleaned up a centrifuge spill of *B. pseudomallei* culture with bare hands (*9*). Symptoms of chills, fever and malaise, tenderness in the right axilla, and pleuritic pain in the right side of the chest developed in the worker 3 days later. The second case-patient was a 33-year-old laboratory worker who performed antimicrobial drug susceptibility testing on 2 apparent *B. cepacia* isolates, 1 of which was actually *B. pseudomallei* isolated from the blood of a 29-year-old Vietnam veteran with recurrent cavitating pneumonia (*10*). Four days later, fever, pleuritic chest pain, a productive cough, and swelling of the right calf developed. Both persons were cured after a prolonged course of antimicrobial drugs. Inhalation of an infectious aerosol was thought to be the likely route of infection in both case-patients, although the first case-patient had an ulcerative lesion at the base of the right index finger at the time of exposure; infection through this lesion cannot be ruled out.

The suggestions in this article have been developed by a group of clinicians and laboratory workers who have many years of experience working with naturally acquired melioidosis and its causative organism. This guidance is applicable to exposure events that have occurred in research or diagnostic laboratories. The scope includes advice to laboratories practicing within areas where melioidosis is endemic, and where serologic investigation of a laboratory exposure event is often complicated by the possibility of prior exposure to an environmental source. Additional guidance is given about laboratory exposure to the closely related organism, *B. mallei*. The guidance does not address actions required after a bioterrorist event. Guidelines for action in the event of a deliberate release have been posted by the United Kingdom Health Protection Agency (www.hpa.org.uk/infections/topics_az/melioidosis/menu.htm).

## Action Required Before Working with *B. pseudomallei*

### Prevention of Laboratory Infection with *B. pseudomallei*

Good laboratory practices will prevent most laboratory accidents involving exposure to *B. pseudomallei*. The organism should be handled by trained personnel within a Biosafety Level 3 (BSL-3) facility (or national equivalent); laboratory practices specified by the respective national legislative and institutional biosafety committees should be used. Laboratory workers should obtain organism- and site-specific training that includes orientation training for new workers and annual refresher training for all workers. Work should be conducted in a biologic safety cabinet and gloves should always be worn when manipulating these microorganisms. Respiratory protection must be used during centrifugation or when handling infected animals. Sealed cups should be used in all centrifuges, and these should be opened only in a biologic safety cabinet. More complete descriptions of safe work practices, personal protective equipment, and engineering controls associated with such laboratories in the United States can be found in Biosafety in Microbiological and Biomedical Research Laboratories (BMBL) (available from www.cdc.gov/od/sap; *11*). Elsewhere, practice should follow relevant national guidelines. General international guidelines are provided by the biosafety manual of the World Health Organization (WHO) (*12*). In the United States, researchers and facilities handling select agents (including *B. pseudomallei* and *B. mallei*) must be registered, inspected, cleared, and approved by the proper federal agencies before they obtain the agents and begin any research.

Clinical diagnostic laboratories functioning at BSL-2 may isolate *B. pseudomallei* from a variety of sample types. In this case, all work should be transferred to appropriate containment facilities as soon as *B. pseudomallei* is suspected, and if the bacterial identity is confirmed the risk of potential exposure to laboratory staff must be assessed. Although diagnostic and research laboratories in resource-poor settings across Asia rarely have access to BSL-3 facilities, such laboratories can adapt many of the practices described here to work in a BSL-2 laboratory for little or no extra cost, and safe laboratory practices will serve to minimize the risk of exposure to laboratory workers. This guidance should act to serve resource-poor laboratories that are in a position to provide life-saving culture results, rather than hinder such activities. However, if the United States provides research funding to a laboratory, regulatory compliance at the local level must conform to US standards.

### Susceptibility Pattern

Work with known antimicrobial-resistant *B. pseudomallei* strains should be minimized unless resistance issues are the focus of the research. It is good practice to establish susceptibility to meropenem, ceftazidime, trimethoprim-sulfamethoxazole (TMP-SMX), doxycycline, and amoxicillin-clavulanic acid for all *B. pseudomallei* isolates in current use in the laboratory. This choice is based on the fact that the last 3 drugs listed may be used for PEP, and ceftazidime or meropenem are the drugs of choice for the initial treatment of melioidosis. This information should be held in a record that is immediately available to safety and medical staff after an exposure event. This is particularly important when working with clinical *B. pseudomallei* isolates from Asia because ≈13% of Thai isolates are resistant in vitro to TMP-SMX, the first-line PEP agent. TMP-SMX susceptibility should be tested by E-test or another reliable MIC-based method; disk testing to determine susceptibility of *B. pseudomallei* to TMP-SMX is unreliable and should not be used (*13*).

### Baseline Serum Sample

Before starting planned work with *B. pseudomallei*, baseline serum samples should be obtained from all workers and logged and stored at –80°C in a secure location. Testing of these samples is only necessary in the event of a subsequent exposure incident. Serum banking must be done in a manner that ensures the privacy of employees and security of the specimens and may in some circumstances need to be contracted to a suitable organization or other laboratory.

### Assessment of Risk Factors

Risk factors associated with *B. pseudomallei* laboratory work are listed in Table 1. Exposure to aerosols represents the greatest biohazard because it can result in inhalation, ingestion, and mucous membrane contact. Assessment of host risk factors for melioidosis in laboratory personnel is based on studies of naturally acquired melioidosis in Thailand and northern Australia. Diabetes mellitus, excessive alcohol consumption, chronic renal failure, and chronic lung disease are independent risk factors (*4*). Persons who are immunocompromised through disease or prescribed drugs (including steroids) are also at increased risk. HIV infection does not appear to constitute a risk factor in northeast Thailand (*14*). Staff with risk factors for melioidosis should be informed of their increased risk. This should be formally explained and documented, and alternative work options should be discussed and provided when requested. In addition to standard precautions and baseline serologic testing, any laboratory staff member working with *B. pseudomallei* who has an identified risk factor should be investigated for *B. pseudomallei* infection if they have a febrile illness, irrespective of history of an exposure event in the laboratory. The physician should arrange serologic testing and appropriate cultures.

**Table 1:** Risk assessment of laboratory incidents involving *Burkholderia pseudomallei*

Low risk
Inadvertent opening of the lid of an agar plate growing *B. pseudomallei* outside a biologic safety cabinet
Inadvertent sniffing of agar plate growing *B. pseudomallei* in the absence of contact between worker and bacterium
Splash event leading to visible contact of *B. pseudomallei* with gloved hand or protected body, in the absence of any evidence of aerosol
Spillage of small volume of liquid culture (<1mL) within a functioning biologic safety cabinet
Contamination of intact skin with culture
High risk
The presence of any predisposing condition without proper personal protective equipment (PPE): diabetes mellitus; chronic liver or kidney disease; alcohol abuse; long-term steroid use; hematologic malignancy; neutropenia or neutrophil dysfunction; chronic lung disease (including cystic fibrosis); thalassemia; any other form of immunosuppression
Needlestick or other penetrating injury with implement contaminated with *B. pseudomallei*
Bite or scratch by experimental animal infected with *B. pseudomallei*
Splash event leading to contamination of mouth or eyes
Generation of aerosol outside biologic safety cabinet (e.g., sonication, centrifuge incident)

### Defining a Laboratory for Serologic Testing

The laboratory safety officer is responsible for determining regional or national laboratories that offer serologic services based on the indirect hemmagglutination assay (IHA) or another validated assay. In the United States, serum specimens should be shipped to the respective state health departments for subsequent testing; contact should be made in the first instance with the Division of Foodborne, Bacterial, and Mycotic Diseases, Bacterial Zoonoses Branch, CDC. This information should be in place as a matter of routine and available in the event of an incident.

### Agreeing on a Protocol

All laboratories where *B. pseudomallei* is known or likely to be handled should have agreed-upon arrangements for the provision of occupational health support. In conjunction with their occupational health colleagues, laboratories should prepare a written protocol detailing how staff will be managed both before and after exposure incidents. This must include arrangements for immediate medical assessment after an exposure. Ideally, the name of a specific physician who is experienced in the treatment of melioidosis (nationally or internationally) and who is willing to be consulted about individual incidents, should be identified in advance.

## Action Required in the Event of Accidental Exposure to *B. pseudomallei*

### Immediately Following Exposure

The site of any contamination or inoculation should be immediately washed with copious amounts of water, followed by an appropriate cutaneous disinfectant according to local policy. The designated safety officer for the laboratory should be informed and involved immediately thereafter. The type of material involved (the bacterial strain, its antimicrobial drug susceptibility, and likely concentration of organisms in the culture) should be noted. High-risk exposure events are inhalation, inoculation (puncture), or aerosols into the eye, but all exposure events should be taken seriously and treated as potentially significant.

### Postexposure Management

Following decontamination, exposed persons must immediately report to the prearranged local hospital or clinic. Protocols to support subsequent actions, including sources of expert advice, should be in place. The exposed worker or the supervisor should describe the species and strain of organism (including its susceptibility pattern, if available), the type of exposure, and other pertinent information to the attending medical staff. The worker should be interviewed regarding drug allergies and current health status including risk factors for (natural) melioidosis. A risk assessment should be carried out to determine whether the laboratory incident poses a low risk or a high risk. The list of incidents provided in Table 1 gives a basis for this distinction, but it may also be necessary to seek expert advice. All workers involved in a high-risk incident should immediately begin PEP. For persons involved in a low-risk incident, the decision to give PEP should be based on the presence of known risk factors for natural melioidosis; persons with known risk factors should begin PEP, whereas persons with no known risk factors should be managed with postexposure monitoring alone.

### PEP

PEP has been recommended previously following laboratory exposure to *B. pseudomallei* (*7*,*8*), although evidence for its efficacy in humans is lacking. The efficacy of PEP using oral TMP-SMX, amoxicillin–clavulanic acid or doxycycline has been evaluated in BALB/c mice infected with aerosolized *B. pseudomallei* (*15*). Antibiotics were administered twice daily, at 0, 10, 24 or 48 h after the bacterial challenge and continued for 10 days. Survival of all animals was observed until 21 days. All PEP was ineffective when initiated 48 h post-challenge, but animals receiving TMP-SMX had a 100% survival rate when the antibiotic was started 0, 10 and 24 h post-infection, and amoxicillin-clavulanic acid was the least effective. Fluoroquinoloes given for 14 days did not provide good postexposure protection (defined in terms of survival rates) to BALB/c mice following subcutaneous inoculation of *B. pseudomallei*, even when treatment was begun 6 hours postchallenge (*16*). Ciprofloxacin and doxycycline given 48 hours before or immediately after intraperitoneal bacterial challenge in a mouse model raised the median lethal dose, but *B. pseudomallei* was recovered from surviving animals, and relapse of infection was observed in some treated animals followed up for 5 weeks (*17*).

All those involved in high-risk incidents and low-risk incidents in those with risk factors for melioidosis should be offered PEP after they receive an explanation of risks and benefits. PEP should be prescribed by a physician who should monitor the person for evidence of adverse drug effects. The risks of PEP are not insignificant and should be balanced against the likely risks of infection in any given incident. Even if PEP is offered, the appropriate choice of antimicrobial agent and optimal duration of therapy are unknown. The following recommendations are based on treatment efficacy of naturally acquired melioidosis. If the organism is susceptible and the patient does not have a documented allergy to it, oral TMP-SMX is the agent of first choice and should be given twice daily at the doses presented in Table 2. If the organism is resistant to TMP-SMX or the patient is intolerant, the choice is between oral doxycycline or amoxicillin–clavulanic acid. Doxycycline has been used previously in a case of laboratory exposure (*7*), and this is consistent with the finding that PEP with doxycycline was more effect than amoxicillin–clavulanic acid in an experimental mouse model (*15*). However, data on the efficacy of doxycycline versus amoxicillin–clavulanic acid used for the treatment of natural infection favors amoxicillin–clavulanic acid. Doxycyline monotherapy is associated with a much higher rate of relapse and treatment failure compared with a conventional oral combination of chloramphenicol, TMP-SMX, and doxycycline for the treatment of melioidosis (*18*). Amoxicillin–clavulanic acid treatment has a lower relapse rate than doxycycline monotherapy for the treatment of melioidosis but a lower cure rate than conventional oral therapy (*19*,*20*), the mainstay of which is TMP-SMX (*1*). The mean incubation period in 1 study of naturally acquired melioidosis in Australia was 9 days (range 1–21 days), but only 25% of patients remembered a specific inoculation event (*2*). The incubation period can be as long as 62 years (*21*). A period of 3 weeks of PEP is suggested by the authors, a duration that represents the consensus of clinicians who are experienced in treating melioidosis in Thailand and Australia. Fluoroquinolones should not be used for PEP because clinical trial evidence indicates that their use in the treatment of melioidosis is associated with an unacceptably high failure (relapse) rate (*22*,*23*).

**Table 2:** Recommended *Burkholderia pseudomallei* postexposure antimicrobial drug prophylaxis

Antimicrobial drug	Dosage	Frequency
Trimethoprim-sulfamethoxazole (TMP-SMX)	2 × 160–800 mg (960 mg) tablets if >60 kg, 3 × 80–400 (480 mg) tablets if 40 kg-60 kg, and 1 × 160–800 mg (960 mg) or 2 × 80–400 (480 mg) tablets if adult <40 kg plus folate 5 mg/d	Every 12 h
Doxycycline	2.5 mg/kg/dose up to 100 mg orally	Every 12 h
Amoxicillin–clavulanic acid	20/5 mg/kg/dose. Equates to 3 × 500/125 tabs if >60 kg, and 2 × 500/125 tabs if <60kg	Every 8 h

### Postexposure Monitoring (PEM)

The exposed worker should be instructed to seek medical attention if he or she becomes ill and to mention the possible exposure event. Most important is early recognition of febrile illness with or without a cough. Self-recording of temperature twice daily for 21 days should be performed for all persons after exposure.

In the event of febrile illness (T >38°C), development of a cough, or progressive inflammation at the site of a known inoculation event, blood cultures (initially 2 sets from different venipuncture sites), sputum culture, throat swab, and urine culture (using selective bacterial culture medium such as Ashdown medium or *B. cepacia* agar [*24*]) should be performed, as well as a chest radiograph.

A sample of serum should be taken on the day of the exposure event (day 1). This specimen is stored and saved for testing in conjunction with a baseline (preemployment) sample and convalescent-phase sample, because the identification of seroconversion requires comparison of paired (acute- and convalescent-phase) serum samples. The blood sampling schedule for serum samples after day 1 should be 1, 2, 4, and 6 weeks. The baseline (preemployment) and first 2 serum samples (day 1 and week 1) should be tested for the presence of antibodies after 1 week and subsequent samples (in parallel with earlier samples) should be tested as they are taken. Seroconversion, with the development of an antibody response, indicates exposure. Although traditionally a 4-fold rise in titer is used to diagnose an infectious disease, any reproducible rise between 2 samples should be used as an indicator of seroconversion resulting from *B. pseudomallei* exposure because an alternative explanation is unlikely. Positive results should be verified by repeating the test.

Some persons with culture-proven melioidosis do not have detectable antibodies, so an exposed or even sick person may have a negative test. In a prospective study of melioidosis patients in Darwin, only 155/275 (56%) of patients had an IHA titer on admission ≥1:40 (*25*). Of those with initial seronegative results, 68% subsequently seroconverted after admission (*25*), emphasizing the importance of serial serologic testing.

Persons who have resided in an area where melioidosis is endemic or who have traveled to such areas may have preexisting antibodies to *B. pseudomallei*. This will be demonstrated by the presence of antibodies in the preemployment sample, and is a common scenario for staff working in laboratories in northeast Thailand. There is no evidence to guide the interpretation of a series of titers after exposure in persons who are already antibody positive for *B. pseudomallei.* A rise in titer is likely to indicate a new exposure, although this may not necessarily be related to the laboratory event. A lack of change in IHA titer in this group should not be assumed to indicate no exposure. Given the complexity of this situation, experts in the field should be consulted in such cases.

The antibody test with which people have most experience in regions where the disease is endemic is the IHA test (*25*–*27*). The results are provided as either negative or in the form of a titer. Titers are determined in 2-fold rising dilutions, usually to a maximal dilution of 1:10, 240. The accuracy of low titers in the assessment of exposure to *B. pseudomallei* is not known. A titer of 20 may indicate exposure, but it may also be a false-positive result, and the IHA should be repeated on further samples in conjunction with careful clinical assessment. A titer of 1:40 has been used previously during the investigation of an accidental laboratory exposure (*7*), and is likely to provide a more robust measure of true exposure. The IHA is complex to perform and should only be undertaken by experienced laboratory personnel. Evaluation of other tests described in the literature for the diagnosis of melioidosis indicates that none perform consistently better than the IHA.

### Management of Seroconversion

If a worker seroconverts after laboratory exposure to *B. pseudomallei*, further clinical evaluation and an extended course of antimicrobial drug treatment are recommended. Given the lack of evidence to guide management of persons under such circumstances, this recommendation is based on expert consensus. A physician should assess the patient and investigate (samples for culture, blood tests, imaging) if clinically indicated. We recommend that in persons who seroconvert but remain asymptomatic and culture-negative, the PEP agent should be continued for a total of 12 weeks; during this time the individual should be monitored for adverse drug reactions and clinical manifestations of melioidosis. Follow-up should be continued after cessation of PEP, the duration of which should be based on clinical judgment.

### Management of Culture-confirmed Melioidosis

Therapy for culture-confirmed melioidosis consists of an intensive phase of intravenous antimicrobial agents that is required for a minimum of 10–14 days, followed by an eradication phase of oral antimicrobial agents that are required to complete a 12–20 week course of treatment, or longer if clinically indicated (*3*). Choice and dosage of antimicrobial agents for intravenous and oral treatment are summarized in Table 3.

**Table 3:** Treatment of melioidosis

Initial parenteral therapy
Ceftazidime 50 mg/kg/dose (up to 2 g) every 6–8 h,* or meropenem 25 mg/kg/dose (up to 1 g) every 8 h*
Duration of therapy a minimum of 10–14 d, and longer (4–8 wk) for deep-seated infection
Oral eradication therapy
Trimethoprim-sulfamethoxazole orally every 12 h 2 × 160–800 mg (960 mg) tablets if >60 kg, 3 × 80–400 (480 mg) tablets if 40–60 kg, and 1 × 160–800 mg (960 mg) or 2 × 80–400 (480 mg) tablets if adult <40 kg; ± doxycycline 2.5 mg/kg/dose up to 100 mg orally every 12 h plus folate 5 mg/d Duration at least 3–6 mo

*Plus trimethoprim-sulfamethoxazole 8/40 mg/kg (up to 320/1,600 mg) every 12 h for treatment of patients with neurologic, prostatic, bone, or joint melioidosis.

## Exposure to *Burkholderia mallei*

Glanders is a disease of equines caused by *Burkholderia mallei*, which appears to be a clone of *B. pseudomallei* that has lost genetic material in association with a host-adapted parasitic existence (*28*). The infection is now rare in horses in most parts of the world, and human infections, which may occur in those in close contact with infected horses, are even more rare. *B. mallei* is designated a select agent by CDC.

*B. mallei* has caused infections in laboratory workers associated with aerosol exposure and cutaneous inoculation which generally results in an acute glanders infection (*29*,*30*).The same primary preventive measures as for *B. pseudomallei* exposure are appropriate to reduce the risk of laboratory exposure to *B. mallei.* Clinical manifestations of glanders in humans are similar to those of melioidosis. Despite slight differences in antimicrobial drug susceptibilities, drug regimens that are effective in human melioidosis (which have been better evaluated than those for glanders) would also be expected to be effective in glanders.

Recommendations for the management of exposure to *B. mallei* are the same as those for *B. pseudomallei*, with 1 important exception. Although serum should be taken and stored, no validated serologic test for human glanders currently exists. In patients with melioidosis, antibodies develop that cross-react with *B. mallei* (*31*). Because the IHA test uses a crude mixture of antigens it is possible that the *B. pseudomallei* IHA may be able to detect seroconversion to *B. mallei*, but this has not been validated and cannot be recommended. Careful postexposure monitoring should be undertaken as described above, and persons who have a fever should be investigated promptly.
